# Genetic Heterogeneity in a Cyclical Forest Pest, the Southern Pine Beetle, *Dendroctonus frontalis*, is Differentiated Into East and West Groups in the Southeastern United States

**DOI:** 10.1673/031.011.11001

**Published:** 2011-08-29

**Authors:** Natalie M Schrey, Aaron W. Schrey, Edward J. Heist, John D. Reeve

**Affiliations:** ^1^ Department of Zoology, Southern Illinois University Carbondale, Carbondale IL 62901; ^2^ Fisheries and Illinois Aquaculture Center, Southern Illinois University Carbondale, Carbondale IL 62901; ^3^ Current address: University of South Florida, Department of Integrative Biology, Tampa FL

**Keywords:** *Dendroctonus*, microsatellites, phylogeographic concordance, southern pine beetle

## Abstract

The southern pine beetle, *Dendroctonus frontalis* Zimmerman (Coleoptera: Curculionidae) is an economically important pest species throughout the southeastern United States, Arizona, Mexico, and Central America. Previous research identified population structure among widely distant locations, yet failed to detect population structure among national forests in the state of Mississippi. This study uses microsatellite variation throughout the southeastern United States to compare the southern pine beetle's pattern of population structure to phylogeographic patterns in the region, and to provide information about dispersal. Bayesian clustering identified east and west genetic groups spanning multiple states. The east group had lower heterozygosity, possibly indicating greater habitat fragmentation or a more recent colonization. Significant genetic differentiation (θ_ST_ = 0.01, *p* < 0.0001) followed an isolation-by-distance pattern (*r* = 0.39, *p* < 0.001) among samples, and a hierarchical AMOVA indicated slightly more differentiation occurred between multi-state groups. The observed population structure matches a previously identified phylogeographic pattern, division of groups along the Appalachian Mountain/Apalachicola River axis. Our results indicate that the species likely occurs as a large, stable metapopulation with considerable gene flow among subpopulations. Also, the relatively low magnitude of genetic differentiation among samples suggests that southern pine beetles may respond similarly to management across their range.

## Introduction

The southern pine beetle, *Dendroctonus frontalis* Zimmerman (Coleoptera: Circling) is an economically important pest species that occurs throughout the southeastern United States, and in Arizona, Mexico, and Central America (Payne 1980). Southern pine beetles are responsible for the destruction of pine forests when and where they occur in large numbers (Price et al. 1992). These insects are known to have epidemic outbreaks, during which they are capable of overcoming even healthy pine trees (Payne 1980). Southern pine beetles can disperse considerable distances, about 1 km (Turchin and Thoeny 1993; Cronin et al. 1999) and have up to six generations per year (Trân et al. 2007). These factors make them difficult pests to control. Current management of southern pine beetle focuses on direct control of infestations and silvicultural treatments that increase the resistance of the tree to beetle attack (Fettig et al. 2007).

Previous genetic research on the southern pine beetle using allozymes has identified genetic differentiation among widely distant geographic samples in North America (Anderson et al. 1979; Namkoong et al. 1979; Roberds et al. 1987). No significant population structure was observed among beetles from five national forests within a 500 km radius in the state of Mississippi at eight microsatellite loci (Schrey et al. 2008). These studies indicate the potential for gene flow at scales of hundreds of kilometers, with significant heterogeneity across the species' range.

This study investigates genetic diversity and spatial genetic differentiation throughout the southeastern United States. The first objective was to provide information about population structure, which may be important for effective management of this pest species. Bayesian clustering was used to identify natural groups of southern pine beetles, and characterize the amount of genetic differentiation among geographic samples. The relationship between geographic distance and genetic differentiation among samples was also investigated. The second objective was to compare the pattern of genetic differentiation for the southern pine beetle to phylogeographic patterns of other species observed in the region.

Concordant phylogeographic patterns have been detected and described among many species in the southeastern United States (Soltis et al. 2006). Several taxa, including plants, mammals, reptiles, fish, and insects are divided into east and west groups at concordant break points (Vogler and DeSalle 1993; Walker and Avise 1998; Soltis et al. 2006; Church et al. 2003). Our southern pine beetle samples were collected from an area spanning the locations of these concordant break points. Thus, it was possible to determine to identify if southern pine beetle genetic differentiation matches previously described patterns.

## Materials and Methods

### Sample collection

**Figure 1.**
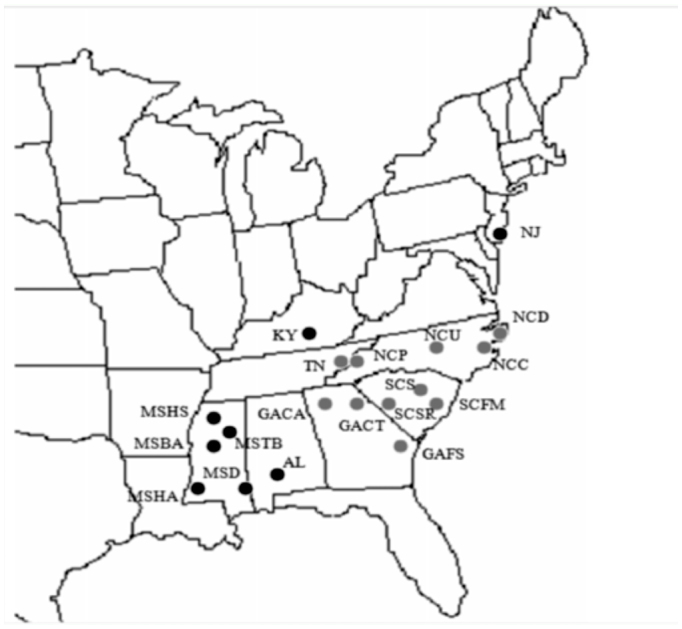
A map of the southeast United States showing sample locations and the two groups defined by Bayesian clustering. High quality figures are available online.

Southern pine beetles (Figure 1; Table 1) were collected during outbreak from fall 2004 to spring 2005 from 19 locations across the southeastern United States using funnel traps (Lindgren 1983) baited with frontalin (PheroTech, Inc., www.contech-inc.com) and turpentine. Traps were placed in 19 locations over eight states and 26 to 100 individuals were screened from each site (Table 1).

**Table 1.**
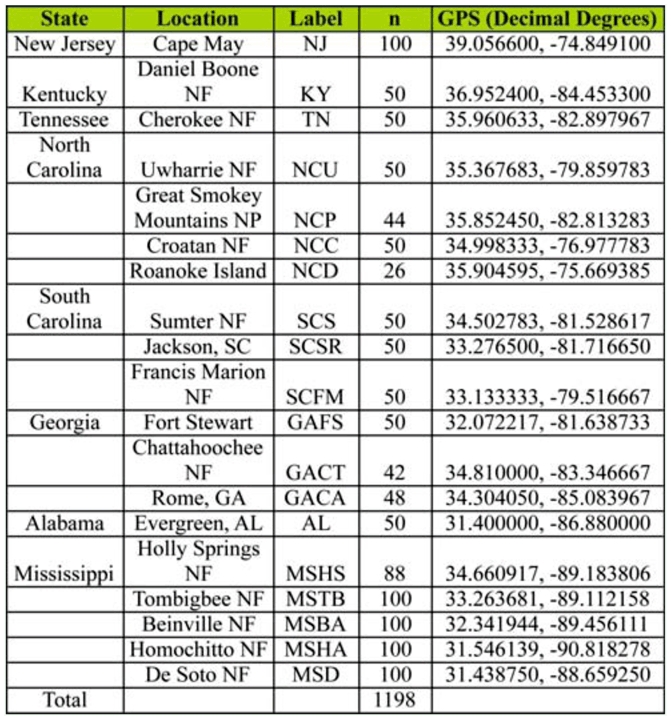
Southern pine beetle collection state, location (National Forest = NF, National Park = NP), label, sample size (n), and GPS for each sample.

Individuals were collected in two locations in New Jersey (3 km apart), Mississippi Holly Springs National Forest (32 km apart), Mississippi Tombigbee National Forest (3 km apart), Mississippi Beinville National Forest (7 km apart), Mississippi Homochitto National Forest (36 km apart), and Mississippi De Soto National Forest (53 km apart). These samples were combined to represent a single location given the lack of genetic differentiation observed at these sites and distances in Schrey et al. (2008). Southern pine beetles were collected from a single site for all other locations. Two of the locations, Tennessee and North Carolina Great Smokey Mountains (NCP), occurred in a continuous forest. The forests in which each site was located are identified in Table 1, but the samples do not represent the entire forest. It is possible that genetic differentiation may occur within a forest.

### Genetic data collection and analysis

Entire specimens were used for DNA extraction with the DNeasy DNA Extraction Kit (Qiagen, www.qiagen.com). Collected individuals (n = 1198) were screened at eight microsatellite loci following the methods detailed in Schrey et al. (2007). Microsatellite loci were briefly amplified by PCR (10 µL final volume), electrophoresed on an ABI 377 (Applied Biosystems, www.appliedbiosystems.com), and genotypes were determined using GENESCAN 3.2.1 and GENOTYPER v 2.5 (Applied Biosystems). Allele size data were binned after visualization on scatter plots. FSTAT version 2.9.3 (Goudet 1995) was used to test each locus in each geographic sample for conformation to Hardy-Weinberg equilibrium and to test all pairs of loci for conformation to linkage equilibrium.

Bayesian analysis of population structure was performed among geographic samples of southern pine beetle using three software packages. First, BAPS version 5.3 (Corander et al. 2008) was used to cluster discrete samples into larger groups with and without geographic data. The presence of 1–27 groups was tested, with the most likely number of genetic groups and the samples constituting each group being identified. Second, TESS version 2.3.1 (François et al. 2006; Chen et al. 2007) was used to characterize population structure among individuals. TESS estimates the number of populations (k) present among individuals and identifies individual membership in each k using a model-based clustering approach. Geographic coordinates were estimated for each individual from the geographic coordinates of each sample location and a pilot analysis was perfomed to confirm that 50,000 sweeps with a 10,000 step burn-in stabilized the likelihood. The preferred k was tested with five runs from k = 2-10. The preferred k was selected by comparing the DIC score and individual assignments. After selecting the preferred k, 100 replicate analyses were run at that k and summarized the runs with CLUMPP (Jakobsson and Rosenberg 2007). For every TESS run, 50,000 sweeps were used with a 10,000 burn-in and a fixed interaction parameter of 0.06 (Chen et al. 2007). Third, the number of genetic groups among all individuals was estimated with STRUCTURE version 2.3 (Pritchard et al. 2000; Falush et al. 2003).The admixture model was used with correlated allele frequencies, 10,000 burn-in steps and 50,000 post burn-in steps. The likelihoods of k = 1–5 groups were determined for four runs at each k by comparing the estimated natural log probability of observing the data (x) given the number of groups, In Pr(x|k). The most likely number of groups was identified by the test that maximizes In Pr(x|k). Individuals were assigned to groups by Q-values, which indicate the proportion of their genotype that originated from each group.

**Table 2.**
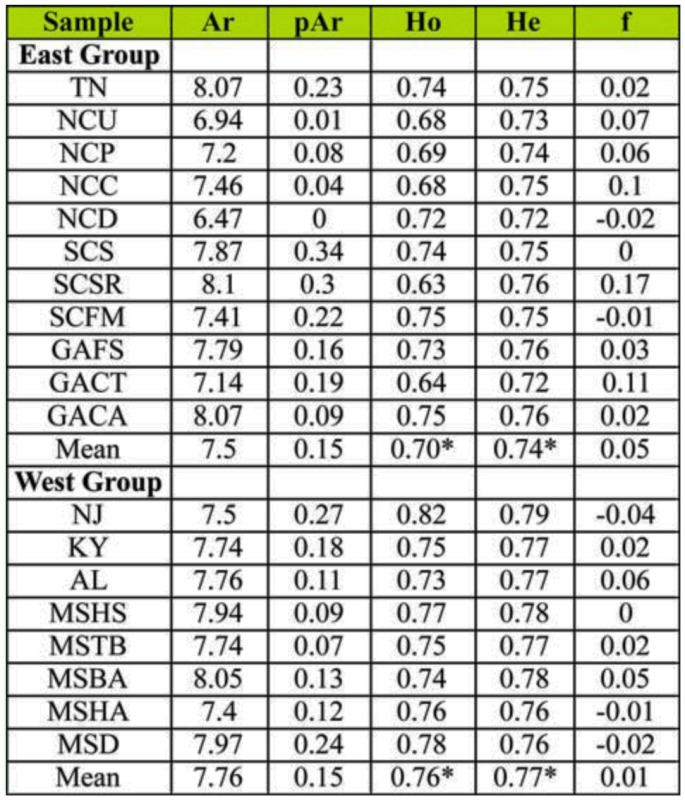
Summary statistics for each sample of southern pine beetle. The allelic richness (Ar), private allelic richness (pAr), expected heterozygosity (H_e_), and observed heterozygosity (H_o_), and the inbreeding coefficient (f) are presented for each sample. Samples have been sorted by Bayesian defined groups and an asterisk indicates significantly different estimates between groups.

The θ_ST_ estimate of F_ST_ (Weir and Cockerham 1984) was calculated among all geographic samples and pairwise among samples with FSTAT. GENALEX-6 (Peakall and Smouse 2006) was used to perform a hierarchical AMOVA to partition genetic variation among samples within Bayesian clustering defined groups Phi_PR_ and Phi_RT_. A Mantel test (Rousset 1997) was performed to compare pairwise genetic differentiation estimates (as OST /(1- θ_ST_)) to pairwise geographic distance (as log_10_ Euclidean distance in meters) with POPTOOLS (Hood 2005). Statistical significance was determined by 999 permutations.

Genetic diversity estimates were calculated for each sample. Allelic richness and private allelic richness were calculated with HPRARE (Kalinowski 2005). Observed heterozygosity, expected heterozygosity, and the inbreeding coefficient were calculated with GENALEX-6. Genetic diversity was compared among geographic samples and among groups defined by BAPS. All statistical tests were corrected for multiple tests using the sequential Bonferroni approach (Rice 1989). T-tests were used to compare genetic diversity estimates among genetic groups defined by Bayesian clustering.

## Results

The microsatellite loci were highly variable. Multiple alleles were observed at each locus (Table 2) and expected heterozygosity ranged from 0.72 to 0.79. Testing Hardy-Weinberg equilibrium found three significant deviations after Bonferroni correction; microsatellite locus Dfr-14 in AL had significantly fewer heterozygotes than expected and microsatellite locus Dfr-24 in NCC and SCS had significantly more heterozygotes than expected. No pair of loci in any geographic sample was significantly out of linkage equilibrium.

**Table 3.**
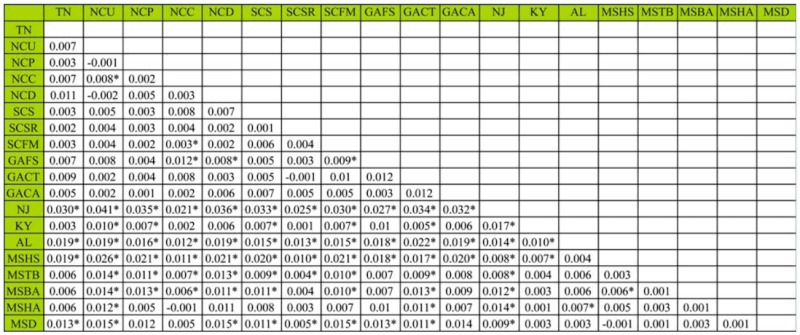
Pairwise θ_ST_ among geographic samples of southern pine beetle. An asterisk indicates statistical significance after Bonferroni correction.

**Figure 2.**
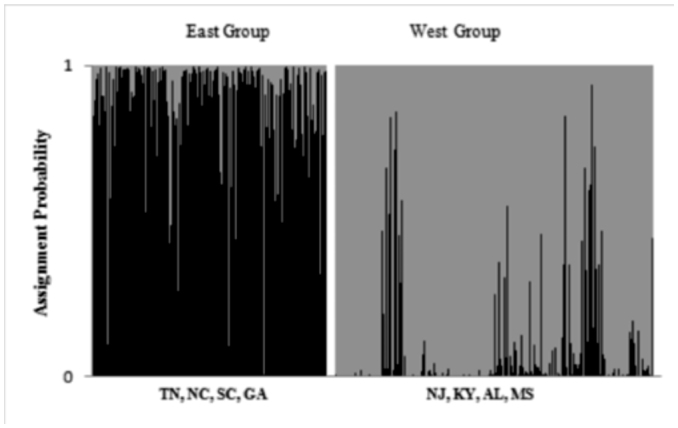
Bayesian clustering of southern pine beetles with the program TESS. Results are provided for 100 runs at k=2 summarized with CLUMPP. Individuals are sorted by sample location and group membership is indicated by color (group 1 = gray, group 2 = black). High quality figures are available online.

Bayesian clustering with BAPS and TESS identified two groups among the geographic samples (Figure 1). BAPS identified the same clustering of samples with and without geographic information. For TESS, the DIC was similar at each k (range 69788 – 69797). However, summarizing 100 runs at k = 2 (Figure 2) clustered individuals into geographic groups concordant with the BAPS analysis. For BAPS and TESS, the southern pine beetle was discriminated into east and west geographic clusters, divided near the Alabama/Georgia state line (Figure 1). The west group included the samples from Mississippi, Alabama, Kentucky, and New Jersey; the east group included Tennessee, North Carolina, South Carolina, and Georgia. The assignments did not perfectly match the geographic distribution of samples. The easternmost sample, New Jersey, was assigned to the western group. Bayesian clustering with STRUCTURE failed to detect multiple groups. The average In Pr(x|k) was 33683 for k(1), -33685 for k(2), -34306 for k(3), -34491 for k(4), and -35277 for k(5).

**Figure 3.**
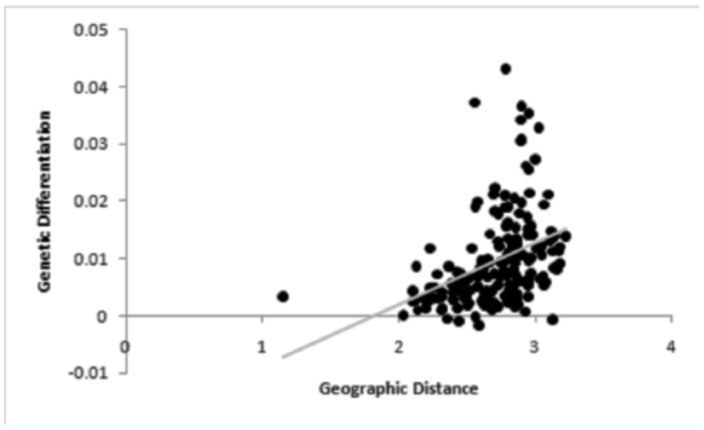
Mantel's test comparing geographic distance, as log_10_ Euclidian distance in meters, to genetic differentiation, as θ_ST_ /(I-θ_ST_), among all samples of southern pine beetle. The Mantel's test identified significant isolation by distance (r = 0.39; P < 0.001). A trendline is provided in gray. High quality figures are available online.

Significant genetic differentiation was observed among samples. OST over all loci and samples was 0.01 (P < 0.0001). Pairwise θ_ST_ values (Table 3) ranged from -0.002 to 0.041, and 79 of 171 comparisons were significant. AMOVA identified slightly more genetic differentiation between Bayesian clustering defined groups (Phi_RT_ = 0.02, *p* = 0.001) than among samples within Bayesian clustering defined groups (Phi_PR_ = 0.01, *p* = 0.001). Within the Bayesian clustering defined east group, 5 of 55 comparisons were significant (Table 3). Within the BAPS defined west group, 11 of 28 comparisons were significant; between the two Bayesian clustering defined groups, 63 of 88 comparisons were significant (Table 3). The New Jersey sample was significantly differentiated from all other samples. The Mantel test was significant (*p* < 0.001) and indicated a positive correlation (r = 0.42) between geographic distance and genetic distance (Figure 3).

Observed heterozygosity and expected heterozygosity were significantly higher in the west group than in the east group (H_o_ t-test *p* = 0.002, H_e_ t-test *p* < 0.001; Table 2). Allelic richness (range 6.47 to 8.10; Table 2), private allelic richness (range 0 to 0.34; Table 2), and the inbreeding coefficient (range -0.04 to 0.17; Table 2) were similar between groups, yet tended to show more alleles, fewer private alleles, and less inbreeding than expected by chance in the west group (Table 2).

## Discussion

The southern pine beetle exhibited genetic differentiation among regions within the southeastern United States, which was weakly compartmentalized into at least two large, multistate groups. Bayesian clustering identified two widespread groups: east samples (Tennessee, North Carolina, South Carolina, Georgia), and west samples (Kentucky, Alabama, Mississippi). The two groups were not geographically congruent. The easternmost samples in New Jersey were assigned to the west group. However, estimates of F_ST_ show that New Jersey samples were differentiated from all other samples. Because of the relatively low level of genetic differentiation observed among most sites, it is possible that the Bayesian clustering methods may have underestimated the true amount of genetic differentiation among locations (Latch et al. 2006). However, two of the three methods used found congruent results among our samples. These methods may have lacked sufficient power to distinguish the New Jersey samples from the remaining samples with Bayesian clustering. If BAPS is forced to form three genetic groups, New Jersey forms an independent group, with the remaining samples assigning identically as with two groups. It is possible that additional samples spanning the range from New Jersey to North Carolina/Kentucky would provide additional information as to the placement of the New Jersey samples.

Observed and expected heterozygosity were greater in the west group than in the east group. Also, allelic richness was slightly higher and private allelic richness was slightly lower in the west group. The greater diversity and fewer private alleles in the west group suggest that this area may have larger, more connected populations that have been longer established. The genetic diversity of the east group would be consistent with smaller, more fragmented populations.

Southern pine beetle genetic differentiation conformed to the previously identified major phylogeographic pattern, which divides the southeast into east and west groups at the Appalachian Mountains/Apalachicola River axis (reviewed by Soltis et al. 2006). Our samples from Kentucky and Alabama were collected from the western edge of the Appalachian Mountains and assigned to the western group. Southern pine beetle genetic structure does not match that of two of its host pine species, the shortleaf pine, *Pinus echinata*, and the loblolly pine, *Pinus taeda* (Al-Rabab'ah and Williams 2002; Xu et al. 2008). The two pine species form east and west groups at the Mississippi River Basin, not the Apalachicola River. Thus, dispersal preferences or different colonization routes near the Appalachian Mountains may cause the genetic structure of the southern pine beetle. Our genetic diversity estimates indicate that southern pine beetles have been established for a longer time west of the Appalachian Mountains, and their dispersal to the east is more recent.

Significant isolation-by-distance, albeit at low magnitudes, was found among southern pine beetles in the eastern United States. Isolationby-distance has been observed in other pine forest beetle species: *Ips confusus* (Cognato et al. 2003), *Dendroctonus ponderosae* (Mock et al. 2007), *Dendroctonus mexicanus* (Zúñiga et al. 2006), and *Tomicus destruens* (Horn et al. 2006). While *Ips typographus* (Sallé et al. 2007) lacked genetic structure in samples collected across Europe, significant genetic differentiation was present among samples from Europe and Asia. Taken together, these studies and our previous study (Schrey et al. 2008) indicate the great potential for gene flow and dispersal or large population sizes slowing genetic differentiation in these insect species. Results indicate that these insects can lack significant genetic differentiation at large-scale distances.

Our results expand the previous genetic studies of the southern pine beetle. The allozyme studies (Anderson et al. 1979; Namkoong et al. 1979; Roberds et al. 1987) showed genetic differentiation among regions at a scale of hundreds of kilometers, and the previous microsatellite study (Schrey et al. 2008) failed to detect significant differentiation among national forests within a 500 km radius. Our results find large multistate/multi-forest groups with slightly higher differentiation between rather than within groups. Isolation-by-distance occurs across the range of the southern pine beetle, with a greater difference occurring between western and eastern samples. The distance required to observe genetic differentiation may be quite large. Thus, southern pine beetles likely lack genetic differentiation within forests and show greater genetic differentiation with increased distance between forests.

Managing the southern pine beetle as a pest has proven difficult because the species is wide-ranging and may exist in large metapopulations. Our results are consistent with southern pine beetle outbreaks originating from geographically proximate individuals. The relatively low estimates of genetic differentiation observed could be caused by gene flow among regions and/or by extremely large populations experiencing low magnitude genetic drift. Evidence for rapid changes in local allele frequencies or widespread significant differences in allele frequencies were not found over short distances. Thus, there does not appear to be large immigrations of beetles from other areas. The lack of genetic differentiation over large geographic areas suggests that successful management practices in one location would be expected to be successful in other locations.
